# Detecting Effects of Low Levels of FCCP on Stem Cell Micromotion and Wound-Healing Migration by Time-Series Capacitance Measurement

**DOI:** 10.3390/s21093017

**Published:** 2021-04-25

**Authors:** Si-Han Wang, Tse-Hua Tung, Sheng-Po Chiu, Hsin-Yi Chou, Yu-Han Hung, Yi-Ting Lai, Yu-Wei Lee, Shiao-Pieng Lee, Chun-Min Lo

**Affiliations:** 1 Department of Biomedical Engineering, National Yang-Ming University, Taipei 11221, Taiwan; d40404006@ym.edu.tw (S.-H.W.); d40004001@ym.edu.tw (T.-H.T.); cindychou2016@ym.edu.tw (H.-Y.C.); ht89127@ym.edu.tw (Y.-H.H.); g39904002@ym.edu.tw (Y.-T.L.); yw30304023@ym.edu.tw (Y.-W.L.); 2 Division of Endocrinology and Metabolism, Department of Internal Medicine, Tri-Service General Hospital Songshan Branch, National Defense Medical Center, Taipei 11490, Taiwan; shengpo.chiu@gmail.com; 3 Division of Oral and Maxillofacial Surgery, Department of Dentistry, Tri-Service General Hospital, Taipei 11490, Taiwan; 4 School of Dentistry, National Defense Medical Center, Taipei 11490, Taiwan; 5 Department of Biomedical Engineering, National Defense Medical Center, Taipei 11490, Taiwan

**Keywords:** ECIS, high frequency, capacitance, mitochondria, micromotion, wound-healing migration

## Abstract

Electric cell–substrate impedance sensing (ECIS) has been used as a real-time impedance-based method to quantify cell behavior in tissue culture. The method is capable of measuring both the resistance and capacitance of a cell-covered microelectrode at various AC frequencies. In this study, we demonstrate the application of high-frequency capacitance measurement (f = 40 or 64 kHz) for the sensitive detection of both the micromotion and wound-healing migration of human mesenchymal stem cells (hMSCs). Impedance measurements of cell-covered electrodes upon the challenge of various concentrations of carbonyl cyanide 4-(trifluoromethoxy)phenylhydrazone (FCCP), from 0.1 to 30 μM, were conducted using ECIS. FCCP is an uncoupler of mitochondrial oxidative phosphorylation (OXPHOS), thereby reducing mitochondrial ATP production. By numerically analyzing the time-series capacitance data, a dose-dependent decrease in hMSC micromotion and wound-healing migration was observed, and the effect was significantly detected at levels as low as 0.1 μM. While most reported works with ECIS use the resistance/impedance time series, our results suggest the potential use of high-frequency capacitance time series for assessing migratory cell behavior such as micromotion and wound-healing migration.

## 1. Introduction

Many animal cell types exhibit the ability to move from one location to another and carry out other subtle motions. Cell motility plays an essential role in physiological and pathological processes such as embryogenesis, wound healing, cancer metastasis, and inflammation [1,2,3,4]. The capacity for cellular motion requires the continuous reorganization and coordinated activity of cytoskeletal, plasma membrane, and adhesion systems [5]. To measure the two-dimensional cell migration of adherent cells, the most common and straightforward approach is using the wound-healing assay. In this method, a wound or gap is created on the surface of a confluent monolayer of cells through mechanical, electrical, chemical, or thermal means, and the wound or gap closure is observed by optical microscopy under a variety of experimental conditions [6]. These visual observations can be graded to provide an estimate of the rate of cell migration. In order to get accurate and reproducible results for this assay, a well-defined procedure to generate a wound of consistent size and measure the rate of closure over time is crucial [6]. Among a variety of wound-healing assays, electric cell–substrate impedance sensing (ECIS) has been developed as an alternative approach to both create the wound and monitor the dynamics of cell migration into the cell-free area in real-time [7]. Furthermore, the ECIS method has been successfully applied to investigate wound-healing migration of various cell cultures including epithelia, endothelia, keratinocyte, and mesenchymal stem cells [7,8,9,10].

ECIS is an impedance-based sensing system that can detect subtle changes between adherent cells and their substrata and hence cell motility [11]. To this end, gold microelectrodes are fabricated on plastic substrata; culture medium is used as the electrolyte [12,13]. An approximately constant and small AC current (~1 microampere) is applied at a specific frequency between a small working electrode and a much larger counter electrode, while both in- and out-of-phase voltages are monitored by a lock-in amplifier. Often, these data are converted to resistance and capacitance considering the cell–electrode system as a series RC circuit. The AC fields normally required for these measurements cause a voltage drop across the cells of only a few millivolts, and no effects of this field or the weak currents on the cells have been detected. This noninvasive measurement is highly sensitive and provides quantitative data regarding cell attachment and spreading, cell morphology, and cell motility [10,14,15]. An important feature of the ECIS is the relatively small size of the working electrodes because cell–substrate interactions are difficult to be detected with larger electrodes. This is a consequence of the constriction resistance through the medium that is much larger than the faradaic impedance of the working electrode and masks fluctuations caused by the adherent cells. When electrodes are reduced to approximately 10^−3^ cm^2^ or smaller, the faradaic impedance predominates, and in this situation, the activities of the adherent cells are sensitively revealed [16,17]. For ECIS wound-healing assay, the elevated AC current (~1.5 milliamperes) is applied for up to tens of seconds, resulting in cell killing. Because of the small electrode size and the close contact of the adherent cells with the electrode, only a few volts will be required to achieve sufficient wounding current across the cells on the electrode surface. The approach is unique in that the same electrodes that supply the wounding currents also serve as a sensing component to follow the subsequent healing process, as neighboring cells migrate from the wound perimeter to fill in the damaged region [7].

Apart from migration or locomotion, cell motility may take the form of continuous shape fluctuations on the nanometer scale, and this subtle aspect of cell motility, or cell body dynamics, is referred to as micromotion [12,18,19,20]. Cellular micromotion was first observed by measuring the electrical impedance of cell-covered electrodes as a function of time [12]. The fluctuation in the impedance time series is due to micromotion and can be related to changes in specific parameters such as cell shape and the distance between the ventral cell surface and the substrate [12]. Other techniques such as quartz crystal microbalance (QCM) and long-range surface plasmon resonance (LRSPR) have also been applied to study cellular micromotion [18,21]. For both methods, the power spectrum of the measured fluctuation data was calculated from Fourier transform and used as a quantitative indicator for comparison. We previously applied ECIS to extensively study cellular micromotion in response to various environmental conditions and demonstrated that it can be an indication of cell metabolism, viability, or motility [10,22,23,24,25]. We also demonstrated that, compared with the average value of the impedance time series, statistical analysis of the impedance fluctuations provides a more sensitive probe. These statistical measures include variance, power spectrum, discrete wavelet transform, as well as the Hurst and detrended-fluctuation-analysis exponents [22,24,25].

In ECIS, the measured impedance always contains resistance (R, the real part of the impedance) and capacitance reactance (X_c_, the imaginary part of the impedance) information and both quantities, R and X_c_, are frequency dependent. The formula for calculating the capacitance (C) from capacitive reactance is: C = 1/2π*f*X_c_, where *f* is the AC frequency in Hertz. Model calculations have shown that cell layer contributions to resistance largely increase at intermediate frequencies (400 Hz–4 kHz) and to capacitance largely decrease at high frequencies (e.g., 40 kHz) [12,14,17]. Recently, we used ECIS to sensitively detect the effect of carbonyl cyanide 4-(trifluoromethoxy)phenylhydrazone (FCCP) on human mesenchymal stem cell (hMSC) micromotion and migration [10]. FCCP is a powerful uncoupler of mitochondrial oxidative phosphorylation. FCCP disrupts ATP synthesis by increasing the proton permeability across the inner mitochondrial membrane and decreasing the proton gradient. FCCP also induces apoptosis if a higher concentration is applied [26]. Although time-series resistances measured at 4 kHz successfully detect the effect of FCCP at concentrations as low as 1 μM, we suspect that capacitance measured at 40 or 60 kHz can do a better job than does resistance. In the present work, we test the feasibility of analyzing the time-series capacitance, measured at 40 or 64 kHz, to obtain micromotion and wound-healing migration data that are correlated with the effect of FCCP on hMSCs. This hypothesis is supported by evidence that high-frequency capacitance readings respond in a linear manner to the fractional electrode coverage by spreading cells [14]. Moreover, while most published papers with ECIS use only resistance time series for data analysis, capacitance measurements at high frequencies have been reported, though rarely, to monitor cell attachment and spreading, proliferation, and wound-healing migration [7,14,15,27,28]. Our results demonstrate a concentration-dependent FCCP-induced decrease in hMSC micromotion and wound-healing migration. The effect of FCCP on hMSCs can be detected at concentrations as low as 0.1 μM. In summary, the present study sensitively quantifies hMSC micromotion and wound-healing migration under the challenge of FCCP with high-frequency capacitance measurement. Capacitance-based detection, therefore, has the potential to provide a sensitive assessment regarding changes in cell morphology and cell migration.

## 2. Materials and Methods

### 2.1. Cell Culture

Human mesenchymal stem cells (hMSCs) were purchased from the Bioresource Collection and Research Center (BCRC, No. RM60596), Hsinchu, Taiwan. ATCC (PCS-500-010). Cells were grown in culture dishes containing 56% Dulbecco’s modified Eagle’s medium (Invitrogen, Carlsbad, CA, USA) and 37% MCDB-201 medium (Sigma Aldrich, St Louis, MO, USA), supplemented with 2% fetal bovine serum (Thermo, Logan, UT, USA), 1× Insulin-Transferrin-Selenium-A (Invitrogen, Carlsbad, CA, USA), 0.5 mg/mL of AlbuMax I (Invitrogen, Carlsbad, CA, USA), 10 ng/mL of epidermal growth factor (PeproTech, Rocky Hill, NJ, USA), 1 ng/mL of platelet-derived growth factor (PeproTech, Rocky Hill, NJ, USA), 10 nM dexamethasone (Sigma Aldrich, St Louis, MO, USA), and 50 μM L-ascorbic acid-2-phosphate (Sigma Aldrich, St Louis, MO, USA) [29]. The cells were maintained at 37 °C in a 5% CO_2_ incubator. When cells reached 70–80% confluence, they were detached with HyQtase (Thermo-Fisher Scientific, Waltham, MA, USA), centrifuged, and resuspended for subculture or further experiments. Cells between Passages 6 and 10 were used in the experiments. Carbonyl cyanide 4-(trifluoromethoxy) phenylhydrazone (FCCP) (Sigma Aldrich, St Louis, MO, USA) was dissolved in DMSO as a stock solution at 10 mM, further diluted in hMSC cultured medium, and then used at various concentrations of 0.1, 0.3, 1, 2, 3, 10, and 30 μM.

### 2.2. Monitoring of Cell Attachment and Spreading

**Figure 1:**
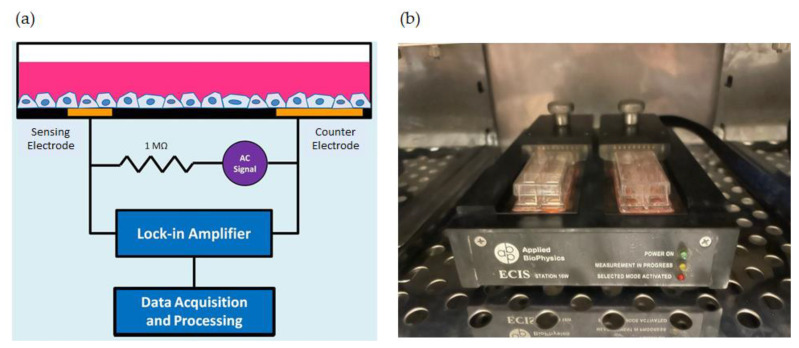
(**a**) Schematic of the ECIS setup. A laptop for data acquisition and processing is connected to a lock-in amplifier, which controls the probe station located inside the incubator. (**b**) Two pairs of 8W1E electrode arrays are connected to the probe station.

Impedance measurements were conducted with an ECIS ZTheta system from Applied BioPhysics (Troy, NY, USA), which is connected to two 8W1E electrode arrays (Figure 1a). Each electrode array contained eight rectangular wells in which a working electrode with a diameter of 250 μm and a much larger counter electrode were made at the base (Figure 1b). The electrode arrays were connected to a lock-in amplifier, which allowed recording in-phase and out-of-phase voltages at 11 different frequencies, ranging from 62.5 Hz to 64 kHz. Cell attachment and spreading were monitored by seeding 4 × 10^4^ cells into each electrode well (1 cm in height, 0.8 cm^2^ in area) for 20 h until the impedance signals became stable, implying the confluency of the cell monolayer was formed. Initial cell density was controlled at about 5 × 10^4^ cells/cm^2^. Cell layers were generally ready for challenge with FCCP in one day. Different concentrations of FCCP solutions were then individually added into each well, and the impedances of all sixteen cell-covered electrodes were monitored for another 20 h. Following that, ECIS micromotion or wound-healing assay was performed to assess the migratory behavior of hMSCs in response to FCCP.

### 2.3. Detection and Analysis of Micromotion

For the detection of cellular micromotion, the impedance of each electrode was measured at 40 kHz every second until 1024 points had been taken. Because of the selection of 40 kHz AC signal for the impedance measurement, the fluctuations in the time-series capacitive reactance were much larger than those in the time-series resistance. Thus, we focused on the capacitance time course for data analysis. The time-series data were normalized and numerically analyzed by calculating Var32, the average variance for the 32-point intervals of the 1024 s data set, as we previously described [22]. Furthermore, discrete wavelet transform (DWT) was used to analyze micromotion data by calculating the following measures of DWT detail coefficients at Level 1: energy, standard deviation (SD), variance (VAR), and signal magnitude area (SMA). Detailed information for calculating these measures of DWT detail coefficients was described in our recent study [24]. In brief, the DWT of a given time-series signal decomposes the signal into different scale components, which represent the same signal but correspond to different frequency ranges. Both detail coefficients (cD) and approximation coefficients (cA) at Level 1, which are the results from the single-level decomposition of DWT, are generated by passing the signal through high-pass and low-pass filters. In this study, Haar (db1) is used as the mother wavelet and detail coefficients are further analyzed as different measures for assessing cellular micromotion.

### 2.4. ECIS Wound-Healing Assay

ECIS wound-healing assay was used to measure the directional migration of hMSCs under the challenge of FCCP. The wounding parameters used to cause the death and detachment of cells on the working electrode were 1.4 mA, 40 kHz, and 10 s. Following wounding, the measured capacitance at 64 kHz increased to a very high value nearing the capacitance of a cell-free working electrode. The capacitances of the working electrodes were continuously monitored for up to 15 h to trace the recovery of the wounded hMSCs under different experimental conditions. To determine the migration rate of hMSCs, the 15 h capacitance data after wounding were selected and replotted as the reciprocal of capacitance versus time. The hill slope was fitted to a sigmoid curve from baseline to the plateau of the reciprocal values of the capacitance time series. The half-time of recovery, T50, was defined as previously described [16,30]. Here, we assume that the recovery rate is inversely proportional to T50.

### 2.5. Mitochondrial Staining and Fluorescence Imaging

hMSCs were seeded on coverslips of 12 mm in diameter for 24 h and then treated with different concentrations (control, 0.1, 0.5, 1, 5 μM) of FCCP for another 20 h. Mitochondria were stained with 100 nM of MitoTracker™ Deep Red FM (Invitrogen, Carlsbad, CA, USA) for 1 h at 37 °C. Cells were then fixed with 4% paraformaldehyde (Electron Microscopy Sciences, Hatfield, PA, USA) for 10 min at room temperature and washed three times with PBS. Slides were examined by epifluorescence microscope with 40× oil objective (Zeiss, Oberkochen, Germany). Images were recorded with a CCD camera and processed with ImageJ.

### 2.6. Statistical Analysis

Results were reported as the mean ± standard error of the mean. A Student’s *t*-test and one-way ANOVA were used to determine the statistical significance of the differences between the control and each of the experimental groups.

## 3. Results and Discussion

### 3.1. Monitoring of hMSC Attachment and Spreading

**Figure 2:**
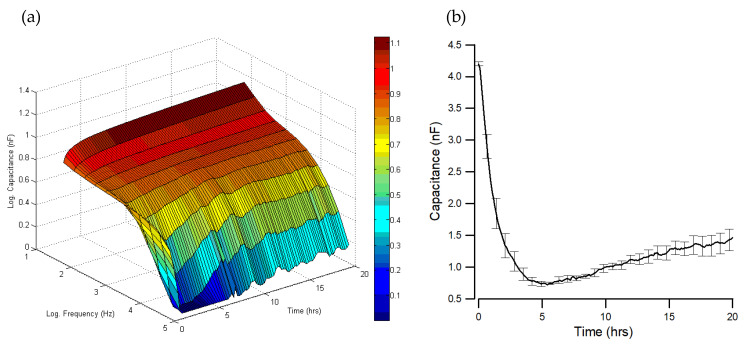
Time-series capacitance measurement of hMSC attachment and spreading. Cells were plated into an ECIS well at time zero, and the seeding density is about 40,000 cells per cm^2^. (**a**) A three-dimensional plot representing the measured time-dependent and frequency-dependent capacitances during the attachment and spreading of hMSCs. (**b**) Time-dependent changes in measured capacitance at 64 kHz during the attachment and spreading of hMSCs. Data obtained from eight working electrodes were averaged and represented as the mean ± standard error of the mean.

To monitor hMSC attachment and spreading, the frequency-dependent capacitances of the cell-covered electrodes were followed at various frequencies. Figure 2a is a typical 20 h three-dimensional graph to display the time-dependent and frequency-dependent changes in measured capacitance. The cell suspension was inoculated into electrode wells at time zero, where the measured capacitance of the cell-free electrode gradually decreased as the applied frequency increased. As cells attached and spread on the electrodes, the measured capacitances at different frequencies varied in different ways. While the measured capacitances at frequencies lower than 4 kHz were only slightly changed at all times, the measured capacitance at a higher frequency (≥4 kHz) significantly decreased with increasing frequency. Specifically, measured capacitances at 64 kHz drastically dropped approximately from 4.2 to 0.7 nF in the first 5 h after seeding (Figure 2b), indicating that high-frequency (e.g., 40 or 64 kHz in the ECIS ZTheta system) capacitance measurement is a very sensitive way to follow cell attachment and spreading on the electrode surface [14]. By the end of 5 h, the capacitance started to increase as the cells started to develop focal adhesions, spread further, and push each other to form a monolayer. The capacitance reached the equilibrium value of ~1.4 nF by the end of 20 h. To understand this result, the interaction of cultured cells with the electrode surface should be considered in detail. When cells attach and spread on the working electrode, the cell bodies effectively block the space available for the movement of ions. If different frequencies of AC signals are employed, the amount of ion current flowing in the space between the ventral side of the cell and the electrode surface, and thus the impedance, will be different. As a result, at a high frequency, less current comes out of the electrode. Moreover, the ion currents do not have enough time to fully charge the surface area of the electrode/electrolyte interface, which is partially or fully covered up by cells [15]. This causes a considerable decrease in the high-frequency measured capacitance, which, as seen in Figure 2b, usually reaches the lowest value four or five hours after inoculation of hMSCs. In our previous study, the resistance spectrum shows that at the high-frequency range (e.g., from 4 to 64 kHz), the resistance increases when cells cover up the electrode area [10]. However, the constriction resistance (the spreading resistance between the sensing electrode and the larger counter electrode) masks the resistance of the cell-covered electrode at a high frequency range [12]. This effect causes the resistance measurement to be less sensitive at 40 or 64 kHz than at 4 kHz for hMSCs.

### 3.2. Capacitance Time Series of hMSCs upon the Challenge of FCCP

**Figure 3:**
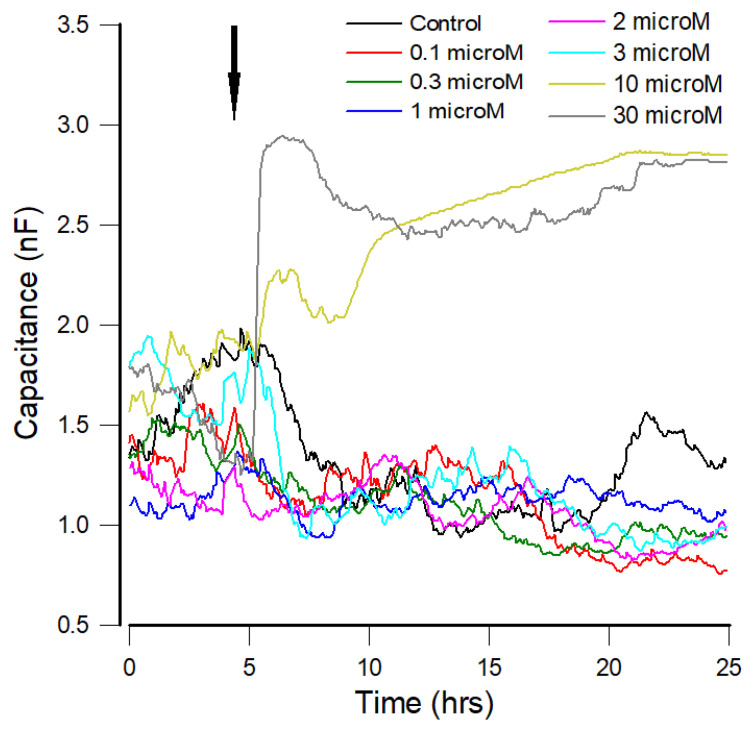
Typical time dependence of the capacitance measured at 64 kHz for hMSCs treated with various concentrations of FCCP. Approximately 20 h after cell seeding, cells developed into confluent monolayers on the working electrodes. At the time point indicated by the black arrow, FCCP was added into wells to give the final concentrations of 0.1 μM (red), 0.3 μM (green), 1 μM (blue), 2 μM (magenta), 3 μM (cyan), 10 μM (yellow), 30 μM (gray), and control (black). The subsequent changes in capacitance measured at 64 kHz were followed for 20 h.

The overall capacitances of the hMSCs after the exposure of various concentrations (0, 0.1, 0.3, 1, 2, 3, 10, and 30 μM) of FCCP were followed for 20 h. Impedance data of the sixteen cell-covered electrodes were successively measured at various frequencies. Because each electrode required 10 s for taking impedance data at 11 different frequencies, the sampling rate for each electrode at each frequency is about 1/160 Hz. Figure 3 displays typical tracings of the measured capacitance at 64 kHz. At low concentrations of FCCP, from 0 to 3 μM, the differences in the average value of the overall capacitance time series were not apparent, implying that hMSC morphology remained fairly stable during the 20 h exposure of FCCP. This result is in accordance with our recent study that in terms of the overall resistance time series monitored at 4 kHz, there are no evident differences among 0, 0.1, 0.3, 1, 2, and 3 μM FCCP-treated hMSCs [10]. The effect of mitochondrial uncouplers such as FCCP is quite dependent on the cell type and the concentration used [31]. For example, pretreatment with 100 nM FCCP for 5 min was reported to significantly improve postischemic functional recovery in the isolated rat ventricular myocytes via a ROS-dependent pathway [32]. In the same study, while 100 nM FCCP caused mitochondrial oxidation with no change in mitochondrial membrane potential, FCCP at 300 nM caused mitochondrial depolarization and exacerbated damage [33]. A low concentration of FCCP at 0.5 μM has been shown to trigger a form of autophagy in 3T3-L1 murine white adipocytes [34]. We recently reported that, in comparison with the control group, both oxygen consumption rate (OCR) and OXPHOS-ATP production of 0.3, 1, 2, and 3 μM FCCP-treated hMSCs decreased with increasing FCCP concentration [10]. However, the overall resistance at 4 kHz in our previous study or the overall capacitance at 64 kHz in this study (as illustrated in Figure 3) was unable to distinguish the effects of 0, 0.1, 0.3, 1, 2, and 3 μM FCCP on hMSCs. As a classical mild uncoupler of OXPHOS, FCCP at concentrations from 0.1 to 3 μM probably did not affect the integrity of hMSC morphology [35].

As shown in Figure 3, at the highest concentration (30 μM), a drastic increase in capacitance was observed almost immediately following the addition of FCCP. This was followed by a slow drop for about 5 h and then a slow increase for about 10 h. The capacitance plateau was ~2.8 nF by the end of 20 h after adding FCCP. At 10 μM, the initial rise in capacitance was less evident, followed by a slow increase, and also stabilized at ~2.8 nF. It was reported that for SH-SY5Y neuroblastoma cells, a low concentration of FCCP (1 μM) caused a complete depolarization of the mitochondrial membrane and modest reduction in intracellular pH without triggering mitochondrial autophagy (mitophagy) [36]. On the other hand, a high concentration of FCCP (10 μM) caused a more profound decrease in intracellular pH that also activated mitochondrial degradation [36]. Furthermore, while 20 μM FCCP was able to induce apoptosis in CCRF-CEM and HL60 cells, 10 μM FCCP was able to induce PC12 cells after 24 h of treatment [37,38]. It was also reported that after 24 h of exposure to 20 μM FCCP, RD cells were elongated, smaller, and attenuated [26]. In addition, treatment with 20 μM FCCP for 48 h was observed to induce apoptosis of ~40% of As4.1 juxtaglomerular cells and human chronic myelogenous leukemia K562 cells [39,40]. Of particular interest is the observation that treatment of HeLa cells with 30 μM FCCP led to a disruption of microtubules and an aggregation of vimentin filaments [41]. The microtubule cytoskeleton has been known to be critical for regulating intracellular architecture and thus for controlling cell spreading [42]. Regardless of different cell types, the addition of 10 or 30 μM FCCP to hMSCs for 20 h significantly increased the capacitance measured at 64 kHz (Figure 3), indicating the decrease of cell coverage on the electrode. Microscopic observation of the electrodes verified that hMSCs contracted and attenuated 20 h after the addition of 10 or 30 μM FCCP (data not shown).

Here, a single open electrode had a capacitance at 64 kHz of about 4.2 nF (C_open_), as shown at hour 0 in Figure 2b. This was the approximate capacitance value for all the working electrodes before cells were seeded into electrode wells. With a confluent layer of hMSCs in place, this value reduced to ~1.4 nF (C_confluent_). The fractional area of the electrode covered with the cell population can be estimated by (C_open_ − C_cells_)/(C_open_ − C_confluent_) [14,15]. Both 10 and 30 μM data (yellow and gray curves in Figure 3) stabilized at ~2.8 nF after 20 h FCCP treatment. The fractional area covered with cells was calculated as (4.2 − 2.8)/(4.2 − 1.4) = 50%, suggesting that high-frequency capacitance measurement can be used to sensitively monitor high concentration FCCP-induced changes in cell morphology.

**Figure 4:**
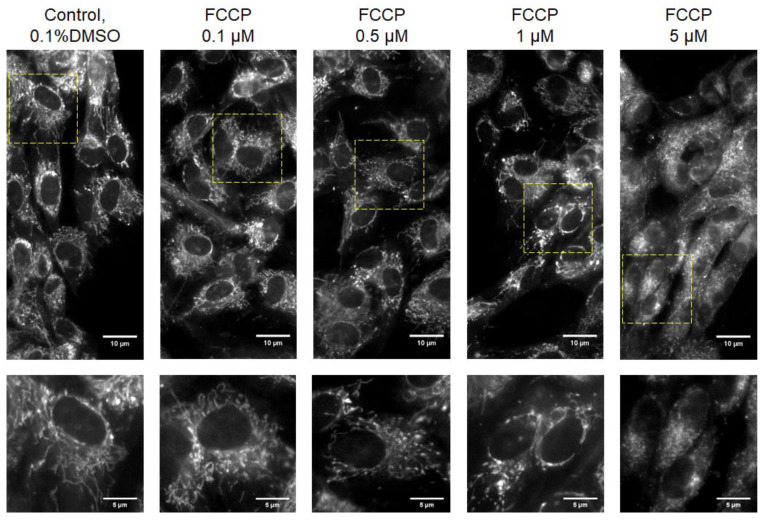
Fluorescence microscopy images of mitochondria from cultured hMSCs, which were under the challenge of various concentrations of FCCP for 20 h and labeled with MitoTracker™ Deep Red FM. Images of the lower panel are enlargements of the areas boxed in the images of the upper panel. Mitochondrial fragmentation was clearly seen for cells treated with 5 μM FCCP.

To further examine the effect of FCCP on mitochondria morphology in hMSCs, mitochondria were stained with MitoTracker™ Deep Red FM visualized by fluorescence microscopy. Figure 4 depicts the mitochondrial morphology after 20 h exposure to different concentrations of FCCP. Overall, in control, 0.1, 0.5, and 1 μM FCCP-treated hMSCs, mitochondria appeared as long and branched structures that spread all over the entire cytoplasm. Disintegration of the tubular network and formation of fragmented mitochondria was clearly observed for cells treated with 5 μM FCCP. This result is consistent with previous findings showing that mitochondrial fragmentation occurred in H9c2 cardiac myoblasts after exposure to 5 μM FCCP or 10 μM CCCP (carbonyl cyanide m-chlorophenyl hydrazone) [43,44]. In these studies, despite significant depolarization of the inner mitochondrial membrane and mitochondrial fragmentation, no disruption of the microtubule network or cellular morphology was observed [44]. Treatment with mitochondrial uncouplers may cause the inhibition of mitochondrial OXPHOS, which are usually associated with the enhancement of mitochondrial fission [45]. It was reported that mitochondrial fragmentation (profission) has been considered an early event during apoptosis [46,47]. Future work is needed to understand whether the impairment of hMSC spreading after exposure to 10 and 30 μM FCCP (as implied in Figure 3) is due to apoptosis.

### 3.3. Capacitance Time Series of hMSC Micromotion upon the Challenge of FCCP

**Figure 5:**
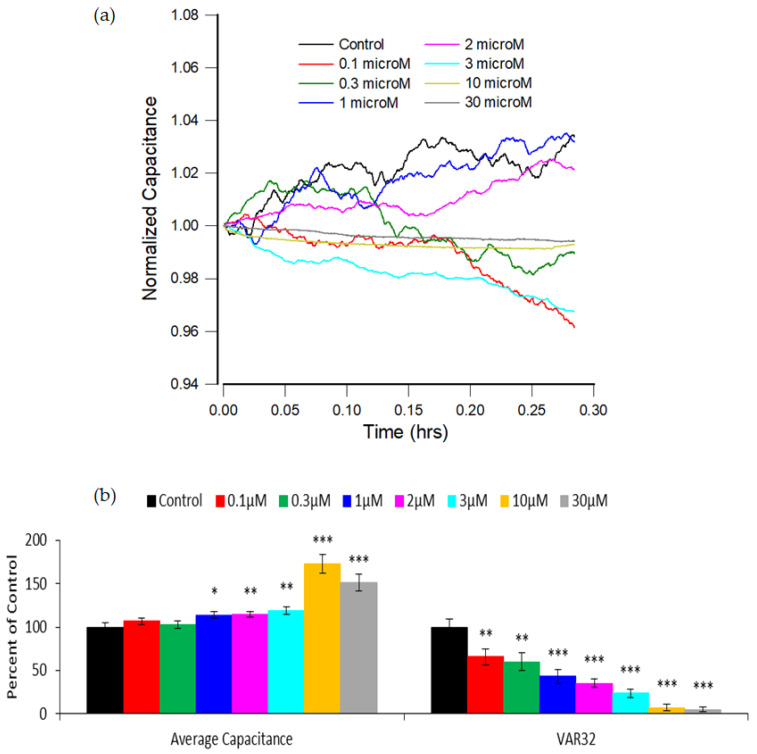
(**a**) Normalized capacitance time series measured at 40 kHz 20 h after the addition of different concentrations of FCCP to hMSC monolayers to make the final concentrations of 0.1 μM (red), 0.3 μM (green), 1 μM (blue), 2 μM (magenta), 3 μM (cyan), 10 μM (yellow), 30 μM (gray), and control (black). Each capacitance time series consists of 1024 data points taken each second, and data were presented by dividing with the first data point. (**b**) Average capacitance value and Var32 analysis over a 1024 s run for each FCCP concentration. For each concentration, at least eight replicate wells from four independent experiments were carried out (n = 8). The results are presented as the percentage of control. Values shown are the mean ± standard error of the mean. * *p* < 0.05, ** *p* < 0.01, *** *p* < 0.001.

In addition to the kinetics of cell attachment and spreading, ECIS is also able to monitor cellular micromotion, which is due to incessant and small changes in cell shape and cell–cell and cell–electrode interactions. These changes can be measured at the nanometer level and are usually correlated with cell metabolic activity [12,22]. In ECIS, micromotion data are quickly recorded as impedance fluctuations of the cell-covered electrodes. As long as the cells are alive, these fluctuations will continue in accordance with the cell type and environmental condition. Figure 5a shows typical micromotion tracings of hMSCs 20 h after exposure to different concentrations of FCCP. At this moment, cells had stabilized and did not display a large downward or upward trend in impedance. These capacitance data were recorded at 40 kHz with a 1 Hz sampling rate and normalized to the values of their starting points, respectively. The reason for normalizing the data curve in this way is to straightforwardly illustrate the relative variations in capacitance time series. Smaller fluctuations were evidently observed for 10 and 30 μM FCCP-treated cells compared with other groups. As shown in Figure 5b, the average capacitance value of the cell layer over a 1024 s run can only distinguish the concentrations as low as 1 μM (*p* < 0.05). It is worth noting that the high average capacitance values of 10 and 30 μM FCCP-treated hMSCs result from the significant decrease of cell coverage on the electrode. Fluctuations in the capacitance time series were also analyzed by calculating Var32 as we previously reported [22,48]. In brief, the normalized 1024 data points were first divided into 32 equal subsets of 32 points each, and the statistical variance of the 32 points in each subset was calculated and averaged for all the subsets to obtain the Var32. As shown in Figure 5b, Var32 displayed a decline in the measures from 0.1 to 30 μM FCCP, and surprisingly, it was able to significantly detect the effect on hMSC micromotion at concentrations as low as 0.1 μM (*p* < 0.01).

Var32 is a simple but effective measure for assessing micromotion data. This measure has been applied to significantly distinguish the cytotoxic effects of cytochalasin B on HUVEC micromotion in a concentration-dependent manner, and the lowest level discernable is 0.1 μM [24,48]. Var32 analysis was also applied to characterize the time courses of overall capacitance measured at multiple frequencies, as shown in Figure 3, where only capacitance data at 64 Hz are presented. However, there were no significant differences among the six capacitance curves measured at low concentrations of FCCP, from 0 to 3 μM (data not shown). The main reason for this incapability is the substantially low sampling rate, ~1/160 Hz, by which 16 cell-covered electrodes are alternately measured at 11 different frequencies. Basically, the higher the sampling rate the measurement has, the more temporal dynamics of the cellular micromotion it can get. It has been suggested in our previous investigation that the sampling rate of the micromotion measurement needs to be 1/16 Hz or higher without losing the discerning power of the Var32 measure [24].

**Figure 6:**
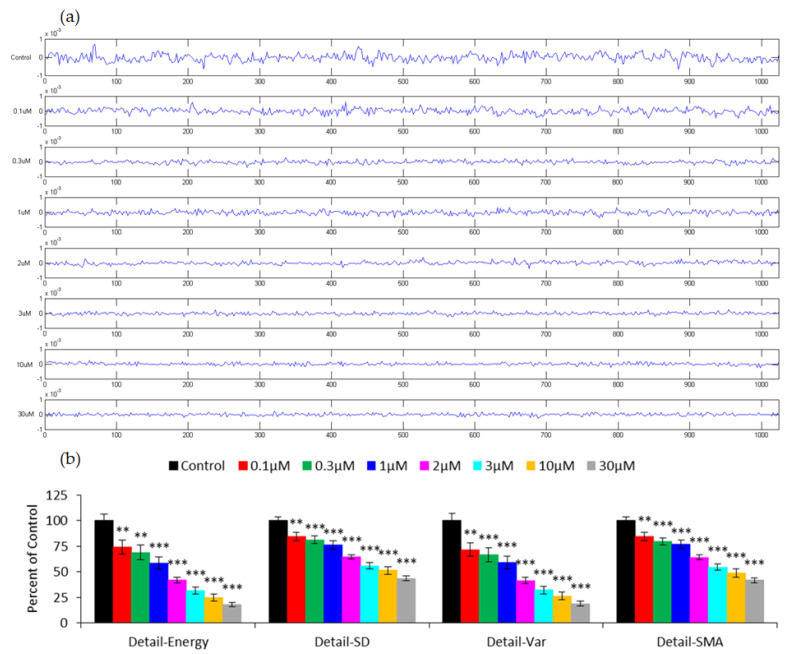
(**a**) Amplitudes of DWT detail coefficients at Level 1 obtained from the time-series capacitance data presented in Figure 5a. Concentrations of FCCP, from top to bottom, are control, 0.1, 0.3, 1, 2, 3, 10, and 30 μM. (**b**) Detail-Energy, Detail-SD, Detail-VAR, and Detail-SMA (signal magnitude area) values of the detail coefficients at Level 1. For each concentration, at least eight replicate wells from four independent experiments were carried out (n = 8). The results are expressed as the percentage of control. Values shown are the mean ± standard error of the mean. ** *p* < 0.01, *** *p* < 0.001.

Discrete wavelet transform (DWT) is a powerful technique in the processing of biomedical signals, which display a broad range of temporal and spectral characteristics. Considering the time-dependent nature of cellular activities, impedance fluctuations obtained from the cell-covered electrodes are associated with micromotion and can be analyzed by DWT. Figure 6a shows the detail coefficients at Level 1 for the capacitance time series presented in Figure 5a, using wavelet Daubechies 1 (db1) as the mother wavelet function. By simply examining the detail coefficients in Figure 6a, it is apparent that there is less fluctuation in the data curve measured at a higher FCCP concentration. The energy, standard deviation (SD), variance (VAR), and signal magnitude area (SMA) of the detail coefficients at Level 1 were calculated, denoted as Detail-Energy, Detail-SD, Detail-VAR, and Detail-SMA, and expressed as a percentage of control (Figure 6b) [24]. For all these measures, a concentration-dependent decrease was generally observed. In addition, the effect of FCCP on hMSCs can be distinguished from the control group at concentrations as low as 0.1 μM (*p* < 0.01), the same discernibility as VAR32 shown in Figure 5b. In contrast to Var32, the main advantage of using DWT for micromotion analysis is that the discerning power stays almost the same even when the sampling rate decreases [24]. With the knowledge of the minimum sampling rate required for recording micromotion data, the maximum number of electrode wells used in each micromotion experiment can be decided.

### 3.4. Capacitance Time Series of hMSC Wound-Healing Migration upon the Challenge of FCCP

**Figure 7:**
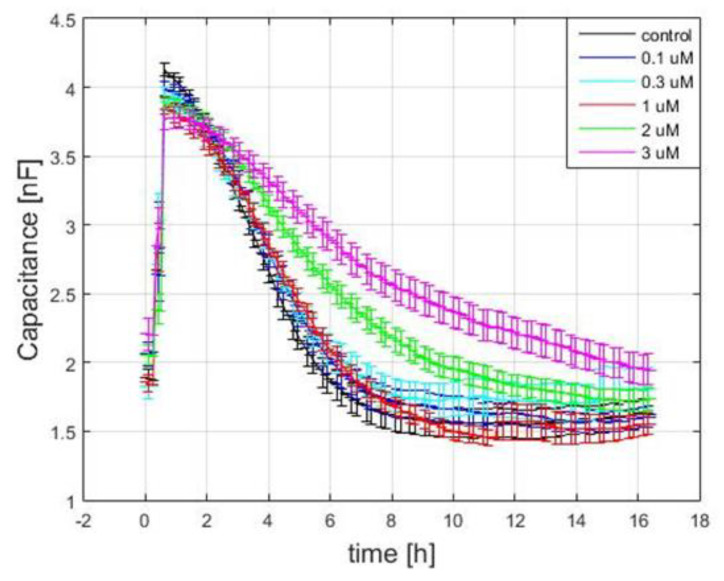
Capacitance time series measured at 64 kHz after electrical wounding of hMSC monolayers. The data were taken 20 h after cells were exposed to 0.1, 0.3, 1, 2, and 3 μM of FCCP and culture medium as control. For each concentration, at least ten replicate wells from five independent experiments were performed (n = 10). Time-series data were averaged and represented as the mean ± standard error of the mean.

**Figure 8:**
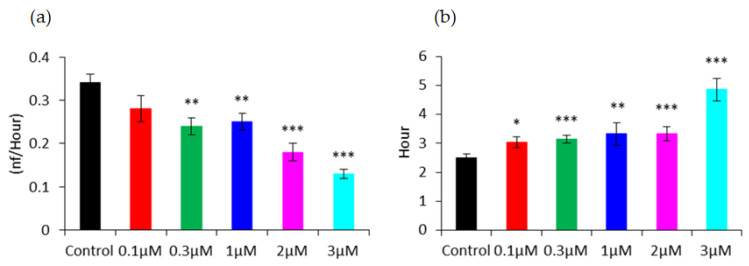
(**a**) Hill slope and (**b**) T50 values calculated from the high-frequency capacitance tracings shown in Figure 7. Values shown are the mean ± standard error of the mean. The statistical significance was calculated by comparing each concentration group with the DMSO control. * *p* < 0.05, ** *p* < 0.01, *** *p* < 0.001.

Similar to the monitoring of hMSC attachment and spreading, high-frequency capacitance measurement can be applied to investigate the effect of FCCP on the wound-healing migration of hMSCs. Cell monolayers were electrically wounded after exposure to various concentrations of FCCP for 20 h. Figure 7 shows the time courses of the capacitance recorded at 64 kHz after wounding. The 15 h recovery curves were then replotted as the reciprocal of capacitance versus time and fitted to a sigmoid curve to obtain both hill slope and T50 measures. As seen in Figure 7, immediately after wounding, the capacitance quickly increased to approximately 4 nF, which is close to the typical capacitance of a cell-free working electrode. Because the working electrodes have the same size, 250 μM in diameter, the time needed for cells to migrate inward from the wound edge until the healing process is completed presumably depends on their migration rate [7]. Because hMSCs were no longer confluent and barely motile after 20 h exposure to 10 and 30 μM, wound-healing migration assay was unable to obtain the recovery data. After hMSCs were treated with 0, 0.1, 0.3, 1, 2, and 3 μM of FCCP for 20 h, both a decrease in hill slope and an increase in T50 were generally observed in a concentration-dependent manner (Figure 8). This result suggests that the migration rate of hMSCs is closely coupled to the mitochondrial oxidative phosphorylation. In addition, by looking at the T50 values, we can detect the effect of the FCCP on hMSCs at levels as low as 0.1 μM, the same discernibility as micromotion analysis displayed in Figure 5 and Figure 6.

Cytoskeletal rearrangement is crucial for cell motility and requires an abundance of ATP. In addition, mitochondrial uncouplers (e.g., FCCP, CCCP, and antimycin A) are associated with a reduction in OXPHOS and thus the inhibition of ATP synthesis, which leads to the disruption of cell motility [31]. It was reported that 10 μM FCCP or CCCP significantly inhibit the movement of cytoplasm organelles in 3T3 cells and chicken neurites [49]. It was also reported that C2C12 myoblasts treated with 2 μM of antimycin A caused a harsh and immediate decrease in oxygen consumption rate (OCR) and a delayed and modest decrease in migration velocity along fiber scaffolds [50]. In view of the central role of ATP in energy supply, it is not surprising that mitochondrial uncouplers are able to disrupt the micromotion and migration of hMSCs. Cellular micromotion and migration, on the other hand, can be potential predictors of hMSC metabolism in response to mitochondrial uncouplers. However, assessing the effect of low concentrations of FCCP (e.g., from 0.1 to 1 μM) on hMSC motility remains challenging. It is noteworthy that in our previous study, the Seahorse XF-24 extracellular flux analyzer was only able to detect the effect of FCCP on both maximal respiration and ATP production of hMSCs at concentrations as low as 2 μM [10]. Furthermore, scratch-wound-induced migration assay showed that the migratory rate of hMSCs was not significantly different between the control group and the cells exposed to 1 μM FCCP for 20 h [10]. In the present work, we applied ECIS to measure time-series capacitance at 40 or 64 kHz and detected the migratory behaviors of hMSCs in response to different concentrations of FCCP. Numerical analysis of both micromotion and wound-healing assays successfully distinguished FCCP levels as low as 0.1 μM, lower than the value (1 μM) obtained from our previous investigation using resistance time course measured at 4 kHz [10]. Capacitance measured at a high frequency (32 kHz or higher) has been suggested as an excellent measure of the cell coverage of the electrode, an indication of the change in cell shape, as well as the damage and recovery of the cell monolayer following injuries [13,16]. Our results confirm the hypothesis that in ECIS high-frequency capacitance measurement provides an exquisitely sensitive assessment of hMSC micromotion and wound-healing migration.

## 4. Conclusions

This study expands and optimizes existing ECIS techniques, as well as demonstrating the possibility of using the high-frequency capacitance measurement with ECIS for both micromotion and wound-healing migration assays. The approach allows for sensitive detection of FCCP-induced decrease in migratory capacity of hMSCs in a concentration-dependent manner. Micromotion and wound-healing migration are two different types of cell movement; however, both assays sensitively distinguish toxin levels as low as 0.1 μМ. Although there is only a single cell type employed in the present work, the measuring techniques and analytical methods used here may be applied to other cell types including endothelia and epithelia. These applications of time-series capacitance measurement with ECIS can be further used to study cytotoxic compounds that affect cell motility or cell body dynamics.

## Data Availability

The data presented in this study are available upon reasonable request from the corresponding author.
